# Correlative study of *Parnassia palustris* extract on orthotopic glioma suppression and gut microbiota modulation in mice

**DOI:** 10.3389/fmicb.2026.1843204

**Published:** 2026-07-31

**Authors:** Ruijun Wang, Huifang Li, Rile Ge, Yuanming Pan, Zhanbiao He

**Affiliations:** 1 Department of Neurosurgery, The Affiliated Hospital of Inner Mongolia Medical University, Hohhot, China; 2 Department of Pediatric Internal Medicine Department, Maternal and Child Health Hospital of Inner Mongolia Autonomous Region, Hohhot, China; 3 Inner Mongolia Medical College Graduate School (Jinshan Campus), Jinshan Economic and Technological Development Zone, Hohhot, China; 4 Cancer Research Center, Beijing Chest Hospital, Capital Medical University/Beijing Tuberculosis and Thoracic Tumor Research Institute, Beijing, China

**Keywords:** glioma, gut microbiota, gut-brain axis, orthotopic model, *Parnassia palustris* Linn (Meihuacao), prebiotic

## Abstract

**Objective:**

The gut microbiota is associated with brain tumor development and prognosis. Natural products are increasingly recognized for their therapeutic potential. Based on previously observed anti-glioma effects of *Parnassia palustris* (MHC) *in vitro*, this study evaluates the efficacy of its oral extract in an orthotopic glioma model and explores the correlative relationship between its antitumor activity and alterations in gut microbiota composition, without inferring causation.

**Methods:**

Four-week-old female Balb/c nude mice underwent intracranial implantation of RFP-U87 MG or U87 MG cells. Mice received intragastric administration of MHC extract (25 mg/kg every other day from day 6 to 24 post-implantation, *n* = 7) or PBS (*n* = 7). Untreated healthy mice served as controls (*n* = 6). Tumor growth was monitored via *in vivo* fluorescence imaging. Brain tissues and gliomas were collected for weighing, H&E, and IHC staining. Fecal samples were collected for gut microbiota analysis using Illumina high-throughput sequencing.

**Results:**

*In vivo* imaging and tumor weight measurements demonstrated that MHC significantly suppressed orthotopic glioma proliferation. H&E staining showed reduced tumor cell density, normalized nuclear-to-cytoplasmic ratio, and decreased nuclear atypia in the MHC group. IHC indicated that MHC inhibited the PI3K/Akt pathway, leading to decreased KI67 and increased p53 expression. MHC did not alter *α*-diversity but modulated the abundance of specific bacterial taxa, notably increasing Muribaculum intestinale, *Bacteroides faecichinchillae*, and *Klebsiella oxytoca*, while decreasing Lachnospiraceae bacterium A4 and Helicobacter japonicus. Compared to healthy controls, tumor-bearing mice exhibited distinct microbial profiles.

**Conclusion:**

MHC extract significantly inhibits orthotopic glioma growth and is associated with compositional changes in the gut microbiota, including enrichment of Muribaculum intestinale and *Bacteroides faecichinchillae*. These correlational findings indicate a potential link between microbial shifts and anti-glioma effects; however, causality remains to be established. Future studies employing fecal microbiota transplantation, antibiotic depletion, or germ-free models are required to determine whether microbiota modulation directly mediates MHC’s antitumor activity.

## Graphical abstract

**Figure:**
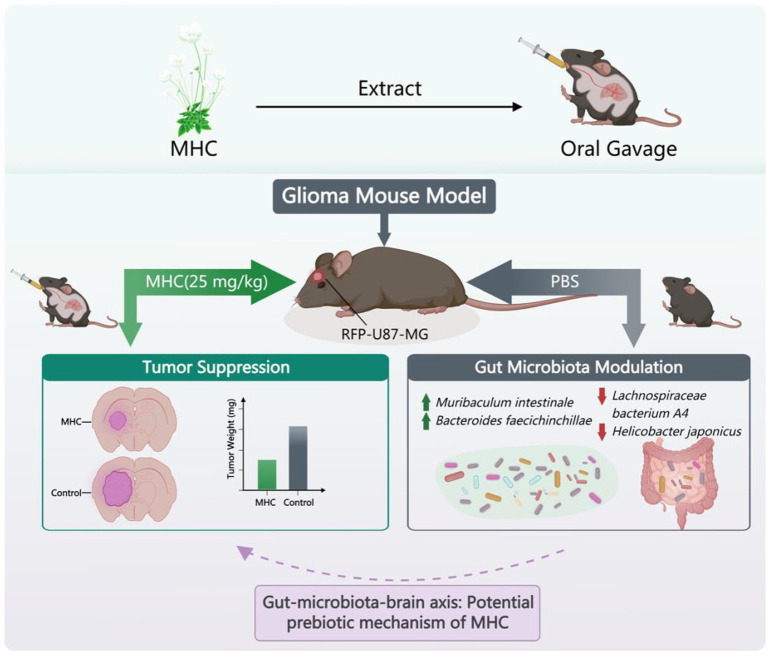

## 1 Introduction

### 1.1 Clinical challenge of glioma and the promise of natural products

Gliomas, which originate from glial cells, represent approximately 40% of all central nervous system (CNS) malignancies and encompass a spectrum from low-grade tumors to the highly aggressive glioblastoma (GBM) (Jayaram and Phillips, 2024; Monje, 2025; Piyadasa et al., 2026; Baker et al., 2025). Despite advancements in surgical resection, radiation, and chemotherapeutic interventions with temozolomide (TMZ) being the standard of care, the prognosis for patients remains dismal due to high recurrence rates, inherent cytotoxicity of treatments, and the development of drug resistance (Li H. et al., 2025; Zhang and Li, 2025; Pu et al., 2025; Yan et al., 2026; Wang et al., 2025). This therapeutic ceiling has driven an urgent search for novel, safer, and more effective anti-glioma agents.

In this context, natural products (NPs) have re-emerged as a pivotal source of anticancer drug discovery. Historically, over 40% of FDA-approved small molecule anticancer drugs are either NP-derived or their derivatives (Zhou Y. et al., 2024; Chaudhry et al., 2022; Naeem et al., 2022; Cui et al., 2025; Hashem et al., 2022). Recent research has consolidated the efficacy of diverse plant-originated small molecules, including curcumin (Soflaei et al., 2018; Shakeri et al., 2019; Arena et al., 2021; Mundekkad and Cho, 2023; Zhao et al., 2024), resveratrol (Liang et al., 2022; Alavi et al., 2021; Kursvietiene et al., 2023; Yang et al., 2022), kaempferol (Amjad et al., 2022; Yang et al., 2023), berberine (Chen G. et al., 2025; Zhu et al., 2022; Zhao et al., 2022; Xu et al., 2025), and their synthetic derivatives, which demonstrate anti-glioma activity through multiple mechanisms such as apoptosis induction, cell cycle arrest, and modulation of key signaling pathways like PI3K/Akt and MAPK (Carnazza et al., 2025; Wang et al., 2022; Zhang et al., 2023; Su et al., 2022). These findings underscore the vast potential of bioactive compounds to offer multi-targeted therapeutic effects with reduced toxicity compared to conventional chemotherapy.

### 1.2 MHC botanical background and medicinal applications

*Parnassia palustris* Linn. (Meihuacao, MHC) is a perennial herb widely distributed across temperate regions of Asia, Europe, and North America. In traditional Chinese and Mongolian medicine, the whole plant has been used for its anti-inflammatory, hepatoprotective, and detoxifying properties (Xin et al., 2019; Chen, 2022, 2023; Zhang et al., 2025; Wang R. et al., 2024, Wang et al., 2026b; Aruhan et al., 2021; Xin et al., 2016; Yan et al., 2016; Hua et al., 2018; Kong, 2025; Yong et al., 2021).

Phytochemical studies have identified several bioactive compound classes in MHC, including flavonoids (e.g., kaempferol and quercetin glycosides), phenolic acids (e.g., gallic acid), iridoid glycosides, and saponins (Aruhan et al., 2021; Xin et al., 2016; Chen, 2022). Among these, flavonoids and phenolic acids are known to possess anti-tumor and immunomodulatory activities, providing a rationale for investigating MHC in glioma models (Kim and Choi, 2021; Reyes-Farias and Carrasco-Pozo, 2019; Jang et al., 2020; Zhou et al., 2023; Chen et al., 2020). The current study focuses on the anti-glioma effects of an MHC extract and the potential involvement of gut microbiota modulation, rather than a comprehensive phytochemical analysis.

### 1.3 MHC mediates the gut-brain axis in neuro-oncology

Returning to the theme of the previous response, the emerging cytotoxic data on MHC invites a speculative but intriguing hypothesis regarding its potential relevance to brain tumor therapy. While the direct pharmacological effects of natural products on tumor cells are well-documented, a paradigm shift is occurring with the growing recognition of the gut-brain axis (GBA) as a critical modulator of brain health and disease (Liu et al., 2022; Chudzik et al., 2021; Dono et al., 2022; Feng et al., 2026; D'Alessandro et al., 2021). The GBA is a bidirectional communication network linking the gastrointestinal tract and the CNS via neural, endocrine, immune, and metabolic pathways (Rusch et al., 2023; Ahlawat et al., 2021; Khan et al., 2025; Chen C. et al., 2025). Central to this axis is the gut microbiome, the “second human genome,” whose composition and metabolic activity profoundly influence CNS physiology, including blood–brain barrier (BBB) integrity, neuroinflammation, and microglial maturation (Ahmed et al., 2022; Barati et al., 2026; Chenghan et al., 2025).

Pioneering studies have established the foundational evidence for microbiota-gut-brain communication. Sudo et al. (2004) first demonstrated that germ-free (GF) mice exhibit hyper-responsive hypothalamic–pituitary–adrenal (HPA) axis reactions to psychological stress, a phenotype reversible by early colonization with *Bifidobacterium infantis*, providing the first direct evidence that commensal gut bacteria influence central stress-response systems. Subsequently, Diaz Heijtz et al. (2011) showed that normal gut microbiota modulates brain development and behavior, with GF mice displaying increased motor activity and reduced anxiety-like behavior. Neufeld et al. (2011) further reported that the absence of gut microbiota alters both behavior and brain gene expression in a region-specific manner, including changes in hippocampal BDNF. Bercik et al. (2011) provided causal evidence that intestinal microbiota can directly affect central BDNF levels and behavior independently of the autonomic nervous system, enteric neurotransmitters, or inflammatory pathways. Collectively, these seminal studies culminated in the formal conceptualization of the “microbiota-gut-brain axis” by Cryan and Dinan (2012) establishing a theoretical framework that has guided subsequent research into the role of the microbiome in CNS disorders, including brain tumors.

Recent evidence implicates the gut microbiome in the pathogenesis and progression of gliomas. Gut dysbiosis, an imbalance in microbial composition, has been associated with chronic inflammation and immune suppression, which are key drivers of tumorigenesis. Mechanistically, gut-derived metabolites such as short-chain fatty acids (SCFAs, e.g., butyrate, propionate) and neurotransmitters (e.g., serotonin, dopamine) can enter the circulation and influence the tumor microenvironment (TME) (Fan et al., 2022; Morad et al., 2025; Zhou M. et al., 2024; Zhang et al., 2025). For instance, SCFAs can modulate BBB permeability and promote an immunosuppressive TME by supporting M2 microglial polarization and regulatory T-cell (Treg) differentiation, thereby facilitating immune evasion. Furthermore, microbial metabolites can activate oncogenic signaling cascades, including the NF-κB, STAT3, and MAPK pathways, which drive tumor proliferation and therapy resistance (Fan et al., 2022; Zhou M. et al., 2024).

However, whether the extracts of MHC could play a prebiotic role in anti-glioma treatment through modification of gut microbiota remains unclear. Our study will systematically investigate the hypothesis that MHC may function as a potential prebiotic, remodeling the gut microbial ecosystem to influence the gut–brain axis, thereby contributing to the suppression of orthotopic glioma development.

## 2 Materials and methods

### 2.1 Cells and cell culture

The GBM cell lines RFP-U87-MG and U87-MG were purchased from the Cell Resource Center, Institute of Basic Medical Sciences, Chinese Academy of Medical Sciences. U87-MG and RFP-U87-MG cells were cultured in high-glucose DMEM complete medium supplemented with 10% FBS and 1% penicillin–streptomycin, in a 37 °C incubator containing 5% CO₂. Three nude mice were used for subcutaneous tumor implantation (bilateral subcutaneous implantation, 1 × 10^6^ cells per site). These tumors were intended for subsequent intracranial tumor implantation via open surgery. Once the subcutaneous tumors reached 8 mm in diameter, they were aseptically excised and cut into 1 mm^3^ fragments, avoiding necrotic areas.

### 2.2 Main drugs and reagents

DMEM high-glucose medium, fetal bovine serum (FBS), and penicillin–streptomycin (Pen Strep) were purchased from Beyotime Biotechnology. HE staining of brain tumor tissues and adjacent normal tissues was commissioned to Wuhan Servicebio Technology Co., Ltd., and imaging was performed using a brightfield microscope. *Parnassia palustris* L. was collected in August 2023 from national nature reserves in Tongliao City, Inner Mongolia, and other locations. The freshly collected medicinal material included only the flowers and stems. The raw herb was identified by Inner Mongolia Medical University as the Mongolian medicinal herb *Parnassia palustris* (known locally as Meihuacao, MHC, Wulidige). The material was cut into sections and air-dried. A 200 g sample of the dried *Parnassia palustris* was subjected to water extraction and alcohol precipitation. The herb was decocted in water three times, each for 1 h. The decoctions were filtered, and the filtrates were combined and concentrated to a specific volume (approximately 1 L). Ethanol was then added to achieve a final ethanol concentration of 60% to remove impurities such as starch. After adding ethanol, the mixture was gently shaken, allowed to stand overnight, and filtered the next day. The filtrate was collected, concentrated, and dried to obtain the water extraction and alcohol precipitation extract of *Parnassia palustris* (MHC), which was used for subsequent animal experiments.

After obtaining the aqueous and organic-phase crude extracts, we did not directly administer the solvent-containing extracts via oral gavage. Instead, all extracts were subjected to low-temperature vacuum drying until completely dried powder solids were obtained. The dried whole plant of *Parnassia palustris* (MHC) was pulverized and passed through a 40-mesh sieve. An accurately weighed amount of the powder was extracted with 8 volumes (v/w) of 95% ethanol under reflux twice, each for 1.5 h. The ethanol extracts were combined, filtered, and concentrated under reduced pressure at 50 °C using a rotary evaporator (RE-2000A, Yarong Biochemical Instrument Co., Shanghai, China) until ethanol-free to obtain the ethanol extract residue. The remaining marc was air-dried to remove residual ethanol, and then extracted with 10 volumes (v/w) of deionized water under reflux twice, each for 1.5 h. The aqueous extracts were combined, filtered, and concentrated under reduced pressure at 60 °C using the same rotary evaporator to an appropriate volume. The ethanol extract residue and the aqueous concentrate were pooled, mixed thoroughly, and placed in a low-temperature vacuum drying oven. The drying process was carried out at 40 °C under a vacuum of ≤0.08 MPa using a vacuum drying oven (DZF-6050, Shanghai Jinghong Experimental Equipment Co., Shanghai, China) until constant weight was achieved (with a difference of less than 5 mg between two consecutive weighings performed at 2-h intervals). The dried product was pulverized, passed through an 80-mesh sieve to obtain the crude extract of *Parnassia palustris* (MHC), which was then sealed in airtight containers and stored at −20 °C until further use.

For animal administration, the dried MHC powder was freshly dissolved in sterile phosphate-buffered saline (PBS, pH 7.4) at the desired concentration immediately prior to oral gavage.

### 2.3 Establishment of an orthotopic glioma model

Four-week-old female Balb/c nude mice were used in two batches of experiments: The first batch was used for *in vivo* imaging of RFP-U87-MG fluorescent tumors to evaluate orthotopic tumor growth and the interventional effect of the *Parnassia palustris* (MHC) extract after oral administration (divided into 2 groups, *n* = 3 per group: MHC group and 1 × PBS group). The nude mice were anesthetized, and their heads were exposed and fixed in a stereotaxic apparatus for coordinate marking. A 1 mm^3^ tumor fragment (derived from the subcutaneous tumors) was implanted into the left frontal lobe of each mouse at the following coordinates: 0.5 mm anterior, 2.0 mm lateral, and 2.0 mm depth. The incision was sutured, and the mice were returned to a suitable environment for continued observation. A small animal *in vivo* fluorescence imaging system (IVIS) was used to dynamically monitor the growth of the orthotopic transplanted tumors in the mouse brains. Intragastric administration commenced on day 6 post-tumor implantation (MHC extract administered every other day at 25 mg/kg for 10 consecutive doses; 1 × PBS served as the control). *In vivo* imaging was performed on days 14 and 24 to observe changes in the size of the intracranial tumors in the nude mice. The second batch was used for gut microbiota analysis (*n* = 7 per group). Intracranial implantation was performed using U87-MG cells, and the intervention method was the same as above. Seven untreated healthy mice from the same batch were used as negative controls.

### 2.4 Immunohistochemistry (IHC)

Following the completion of the *in vivo* treatment period, brain tissues were collected, fixed in 4% paraformaldehyde for 24 h, and embedded in paraffin. Coronal sections of the brain were cut at a thickness of 4 μm. For immunohistochemical staining, sections were deparaffinized in xylene and rehydrated through a graded ethanol series. Antigen retrieval was performed by heating the sections in sodium citrate buffer (10 mM, pH 6.0) using a microwave. Endogenous peroxidase activity was blocked by incubation with 3% hydrogen peroxide for 10 min at room temperature. The sections were then blocked with 5% normal goat serum to prevent non-specific binding.

The sections were incubated overnight at 4 °C with primary antibodies against Ki67 (Cat: AF1738, Beyotime Co., LTD, China. Dilution: 1:100), p53 (Cat: AF0255, Beyotime Co., LTD, China. Dilution: 1:200), p-PRAS40 (Thr246, Cat: 44-1100G, Invitrogen., Ltd., US. Dilution: 1:100), p-S6K (Thr389, Cat: ab60948, Abcam., LTD, US. Dilution: 1:50), and p-AKT (Ser473, Cat: 700392, Invitrogen., LTD, US. Dilution: 1:100). After washing with PBS, the sections were incubated with a horseradish peroxidase (HRP)-conjugated secondary antibody for 1 h at room temperature. The immunoreactive signals were visualized using 3,3′-diaminobenzidine (DAB) as the chromogen, and the sections were counterstained with hematoxylin. For negative controls, the primary antibody was omitted.

Stained sections were imaged using a light microscope. For quantitative analysis, five random fields from each section were captured at ×200 magnification. The expression levels of Ki67, p53, p-PRAS40 (Thr246), p-S6K (Thr389), and p-AKT (Ser473) were evaluated by measuring the integrated optical density (IOD) using ImageJ software.

### 2.5 Fecal sample collection and sequencing

Experimental animals from the second batch were euthanized using CO₂. Fresh colonic fecal samples were collected from the nude mice, as well as from the untreated healthy control mice. These fresh fecal samples were placed into sterile collection tubes free of DNA/RNA enzymes, transported on dry ice to Beijing QuantHealth Technology Co., Ltd., and subjected to high-throughput Illumina metagenomic sequencing.

### 2.6 High-throughput sequencing data information analysis

All raw metagenomic sequencing data underwent quality control using MOCAT2 software. Cutadapt software (v1.14, parameter: -m 30) was used to remove adapter sequences from all raw sequencing reads. Subsequently, the SolexaQA package was used to filter out reads with a quality score below 20 and a length less than 30 bp, yielding clean reads. Finally, the filtered reads were aligned to the host genome using SOAPaligner (v2.21, parameters: -M 4 -l 30 -v 10) to remove contaminating host reads, obtaining high-quality clean data.

### 2.7 Microbial community composition analysis

MetaPhlAn3 was used to align the clean reads to clade-specific markers, thereby obtaining the relative abundance of the sample microbiota at various taxonomic levels from species to phylum. Subsequent analyses included species diversity analysis (*α*-diversity), dimensionality reduction clustering analysis (NMDS, PCoA), and inter-group difference analysis (LEfSe).

### 2.8 Statistical methods

SPSS 27.0 software was used for data analysis. Data from repeated experiments are expressed as mean ± standard deviation (SD). Comparisons among multiple groups were performed using one-way ANOVA, and pairwise comparisons between two groups were conducted using the LSD-*t* test. A *p*-value < 0.05 was considered statistically significant.

## 3 Results

### 3.1 Oral administration of *Parnassia* extract suppresses malignant proliferation of orthotopic brain glioma

**Figure 1:**
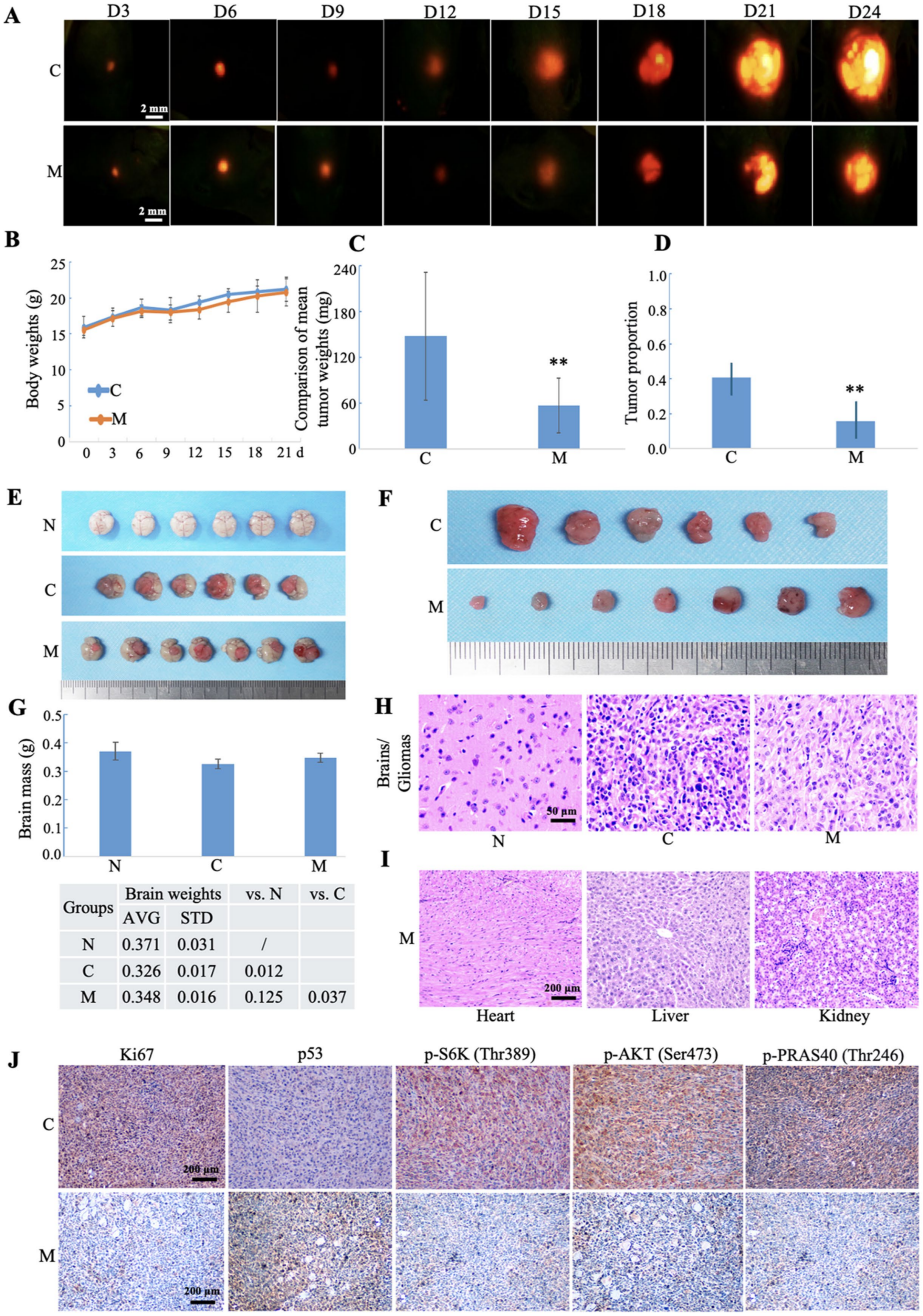
Inhibitory effect of MHC extract on orthotopic RFP-U87-MG glioma. **(A)** *In vivo* fluorescence imaging on day 24 comparing intracranial tumor growth in nude mice from MHC-treated group (M) and the control group (C) (red regions indicate tumor foci). **(B)** Body weight comparison between MHC-treated group (M) and control group (C), showing no statistically significant difference. **(C)** Tumor tissue weight comparison between the two groups, revealing a statistically significant reduction in the MHC-treated group (^**^p < 0.01). **(D)** Tumor proportion between the two groups after dissection, with significant difference observed (^**^p < 0.01). **(E)** Brain mass comparison in the two groups after dissection, with significant difference observed (^**^p < 0.01). **(F)** Tumor tissues in different groups with significant difference. **(G)** Brain mass comparison in the two groups after dissection with significant difference (*p* < 0.05). **(H)** Hematoxylin and eosin (H&E) staining of orthotopic glioma tissues and normal brain tissues. In the control group (C), tumor cells exhibited enlarged, hyperchromatic nuclei and an abnormal nuclear-to-cytoplasmic ratio, whereas MHC-treated group (M) showed a relatively normalized nuclear-to-cytoplasmic ratio and reduced tumor cell density (magnification: 400×, scale bar = 50 μm). **(I)** After oral administration of MHC by gavage, H&E staining of mouse heart, liver, and kidney tissues showed no toxic or side effects on these vital organs (magnification: 100×, scale bar = 200 μm). **(J)** In the two model groups, IHC staining comparison of the cell proliferation-associated protein Ki67, the apoptosis protein p53, and the phosphorylation levels of key proteins in the PI3K/AKT signaling pathway indicated that MHC extract significantly inhibited PI3K/AKT signaling pathway, suppressed tumor cell proliferation, and promoted apoptosis. (Magnification: 100×, scale bar = 200 μm).

To evaluate the therapeutic efficacy of Parnassia extract (MHC), an orthotopic glioma model was established by intracranial implantation of RFP-U87-MG red fluorescent protein-labeled tumor cells. Tumor progression was monitored via *in vivo* fluorescence imaging. Figure 1A illustrates the dynamic changes in tumor size between the two groups with fluorescence signals detection. The MHC extract gavage group showed no substantial effect on the body weight of mice, and there was no statistically significant difference between the two groups (Figure 1B). After dissecting the brain tissues, we observed macroscopic differences between the brain tissues and the orthotopic red gliomas across the different groups. Following weighing of the brain tissue, the glioma tissue was dissected, photographed, and weighed to calculate the tumor weight ratio and compare tumor weights among groups (Figures 1C–G). The results indicated that oral administration of MHC extract significantly reduced malignant proliferation of orthotopic brain tumors. The marked difference in tumor weight was evident: the mean tumor weight in the MHC-treated group (M) was 65.3 ± 21.4 mg, compared to 145.8 ± 67.2 mg in the control group (C) (*p* < 0.01, Figures 1C,F). Further H&E staining revealed an abnormal nuclear-to-cytoplasmic ratio with enlarged and hyperchromatic nuclei in the control group (C) (Figure 1H). Immunohistochemistry showed high expression of the cell proliferation-associated protein Ki67 and weak expression of p53 in the control group, indicating active tumor proliferation. In contrast, the MHC intervention group displayed sparse tumor cell distribution with relatively normalized nuclear-to-cytoplasmic ratios and exhibited significantly decreased Ki67 expression and increased p53 expression, suggesting inhibition of tumor growth (Figure 1J). And there is no obvious toxicity in the important organs including heart, liver, and kidney after MHC treatment (Figure 1I). Based on our previously published findings, MHC extract was shown to affect the PI3K/AKT signaling pathway at both the network pharmacology analysis level and the cellular level, thereby influencing malignant cell proliferation. To further validate this, we assessed the expression of pathway-related proteins in tumor tissues in an *in vivo* model. It was observed that the expression of p-AKT (Ser473), p-S6K (Thr389), and p-PRAS40 (Thr246) proteins was significantly reduced following MHC treatment, indicating that MHC extract inhibits malignant proliferation of tumor cells in vivo through the PI3K/AKT pathway (Figure 1J). Collectively, these results provide compelling *in vivo* evidence that MHC extract exerts its anti-tumor effects by suppressing the activation of the PI3K/AKT signaling pathway in an orthotopic glioma model.

### 3.2 Effect of *MHC* extract on gut microbiota composition

**Figure 2:**
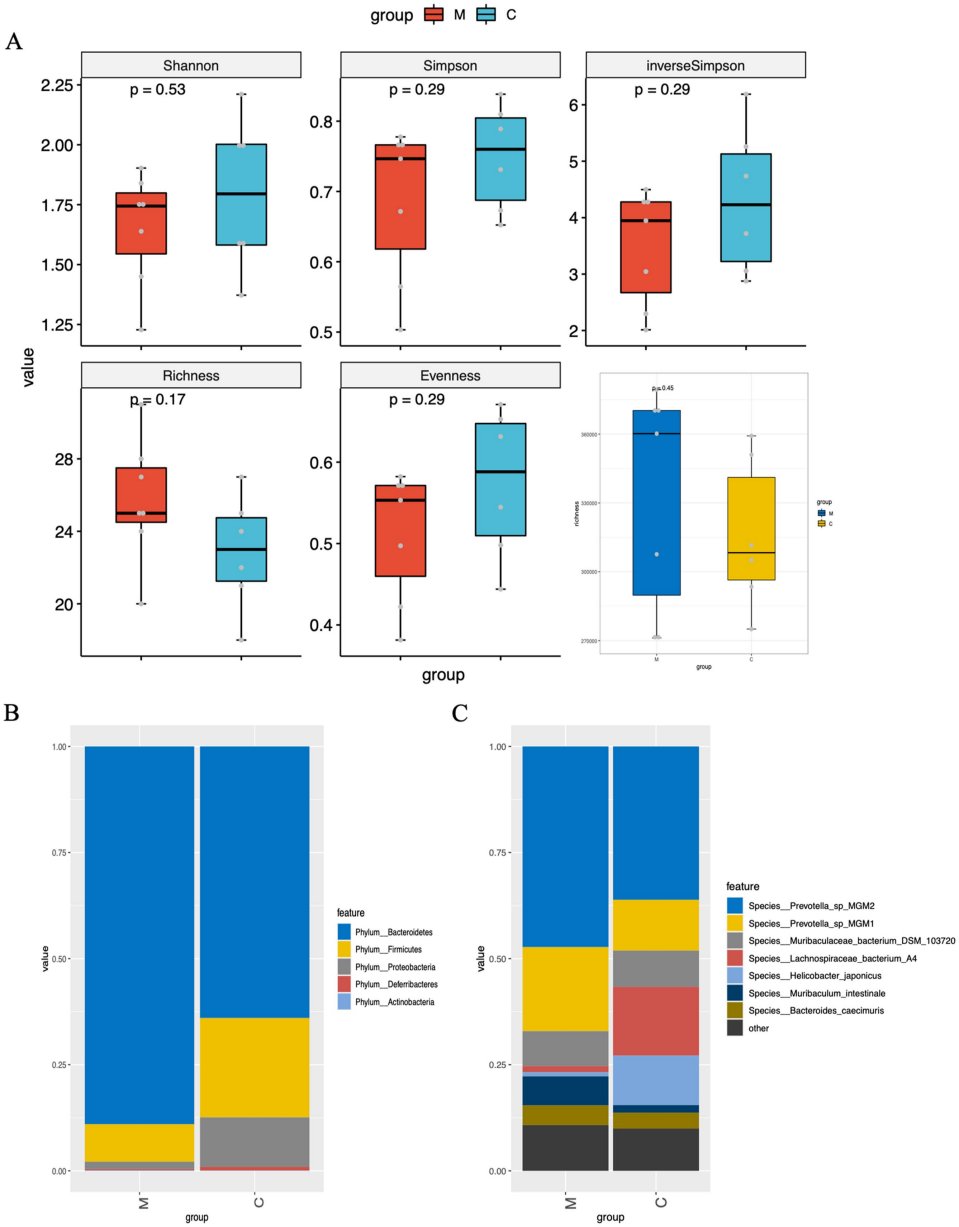
Effect of *MHC* extract on gut microbiota composition in an orthotopic glioma model: **(A)** Comparison of alpha diversity indices (Shannon, Simpson, Inverse Simpson, Richness, and Evenness) between groups, showing no statistically significant differences. **(B)** Relative abundance of gut microbiota at the phylum level, revealing distinct compositional shifts between groups. **(C)** Relative abundance of gut microbiota at the species level, demonstrating significant differences in microbial profiles following *MHC* treatment.

To further investigate the impact of MHC extract on the gut microbiota, two groups of orthotopic glioma-bearing mice (*n* = 7 per group) were established. Mice received intragastric administration of MHC extract (M) or 1 × PBS as a control (C) every other day for 24 days. During gavage administration and in vivo observation of tumor growth, one nude mouse in the control group died prematurely due to rapid tumor progression, whereas no deaths occurred in the MHC treatment group throughout the entire observation period. Fresh fecal samples were collected at the end of the treatment period and subjected to Illumina high-throughput sequencing. Bioinformatic analysis and data read cleaning revealed no significant alterations in alpha diversity indices, including Shannon, Simpson, Inverse Simpson, Richness, and Evenness between the two groups (*p* > 0.05, Figure 2A).

At the phylum level, distinct shifts in microbial relative abundance were observed following MHC treatment, characterized by an increased abundance of Bacteroidetes and decreased abundances of Firmicutes and Proteobacteria (Figure 2B). At the species level, significant differences were also evident: treatment with MHC extract resulted in elevated abundances of *Muribaculum intestinale*, Prevotella sp. MGM2, and Prevotella sp. MGM1, alongside reduced abundances of Lachnospiraceae bacterium A4 and Helicobacter japonicus (Figure 2C).

### 3.3 Effect of *MHC* extract on gut microbiota composition in an orthotopic glioma model

**Figure 3:**
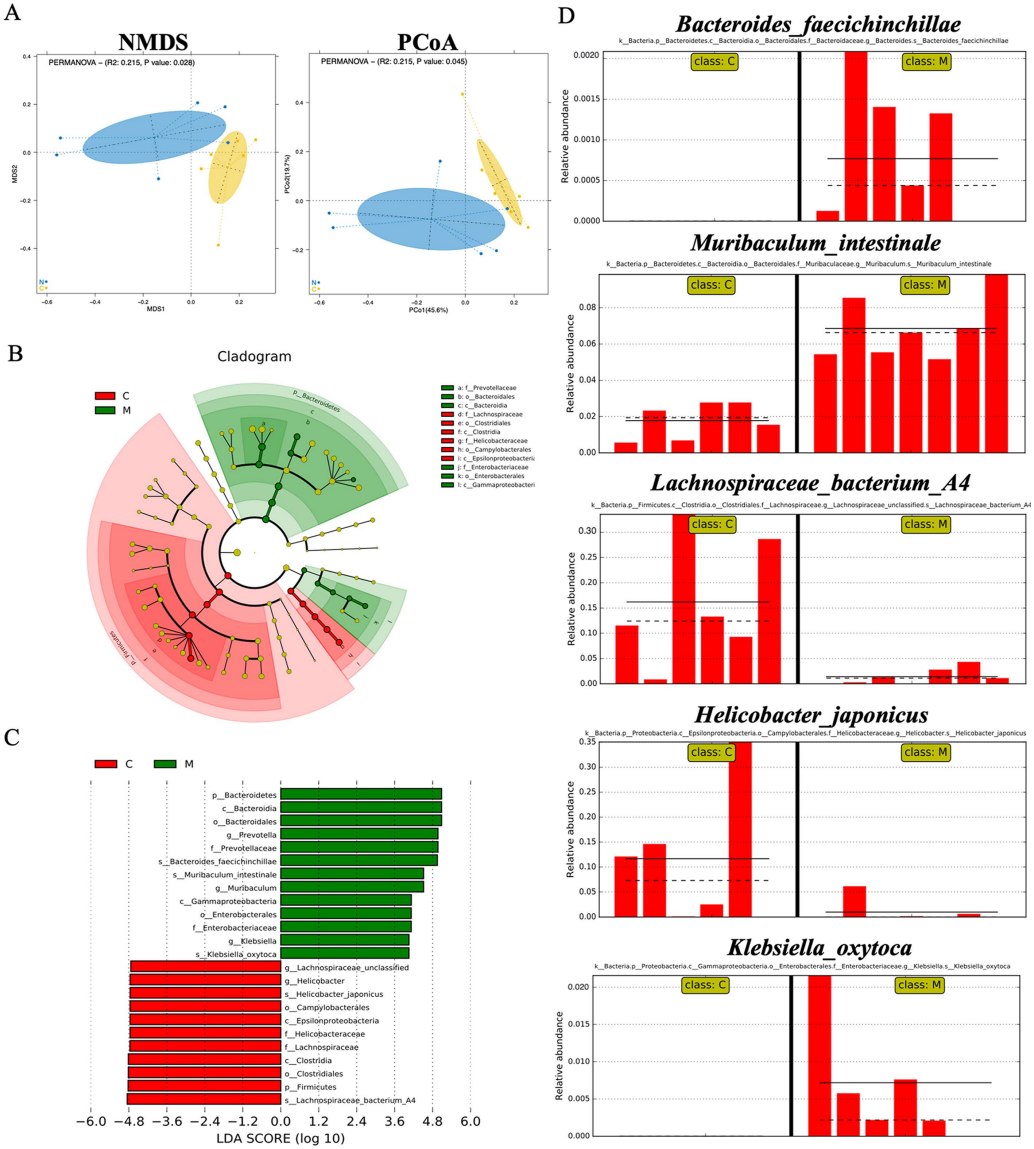
Differential analysis of gut microbiota composition between C and M groups **(A)** Dimensionality reduction analyses based on original species abundance data, including Non-Metric Multidimensional Scaling (NMDS), and Principal Coordinates Analysis (PCoA), revealing significant separation between the two groups. **(B)** Comparison of LEfSe (Linear Discriminant Analysis Effect Size) cladograms between groups, illustrating taxonomic representation of differentially abundant bacteria. **(C)** Histogram of LDA scores from LEfSe analysis, showing the magnitude of effect size for differentially abundant taxa between groups. **(D)** Comparative analysis of the relative abundance of specific differentially enriched bacterial species between the two groups.

To assess the impact of MHC extract on gut microbial community structure, beta diversity analyses were performed using multiple complementary dimensionality reduction approaches. Principal Coordinates Analysis (PCoA), and Non-Metric Multidimensional Scaling (NMDS) were applied to visualize microbial compositional differences between groups. These methods extract principal feature vectors from the microbial abundance data, generating spatial coordinates that reflect the similarity or dissimilarity among samples. In Figure 3A, each symbol represents an individual mouse, with distinct colors indicating treatment groups (Control vs. MHC-treated). The distance between symbols is inversely proportional to the similarity of microbial composition: smaller distances indicate higher compositional similarity and lower dissimilarity. NMDS projects samples into multidimensional space based on species abundance information, with inter-sample distances reflecting the degree of compositional difference. Consistent results were obtained from PCA and PCoA analyses (Figures 3A, *p* < 0.05), collectively demonstrating that MHC intervention significantly altered the beta diversity of gut microbiota in nude mice.

To identify specific microbial taxa with statistically significant differences between groups, Linear Discriminant Analysis Effect Size (LEfSe) was performed (Figure 3B). The LDA score distribution histogram illustrates the magnitude of effect size for taxa showing significant abundance differences between groups, with bar length representing the influence of each differentially abundant species (Figure 3C). Notably, MHC extract treatment significantly increased the relative abundance of *Muribaculum intestinale*, *Bacteroides faecichinchillae*, and *Klebsiella oxytoca*, while decreasing the abundance of Lachnospiraceae bacterium A4 and Helicobacter japonicus (Figure 3D).

**Figure 4:**
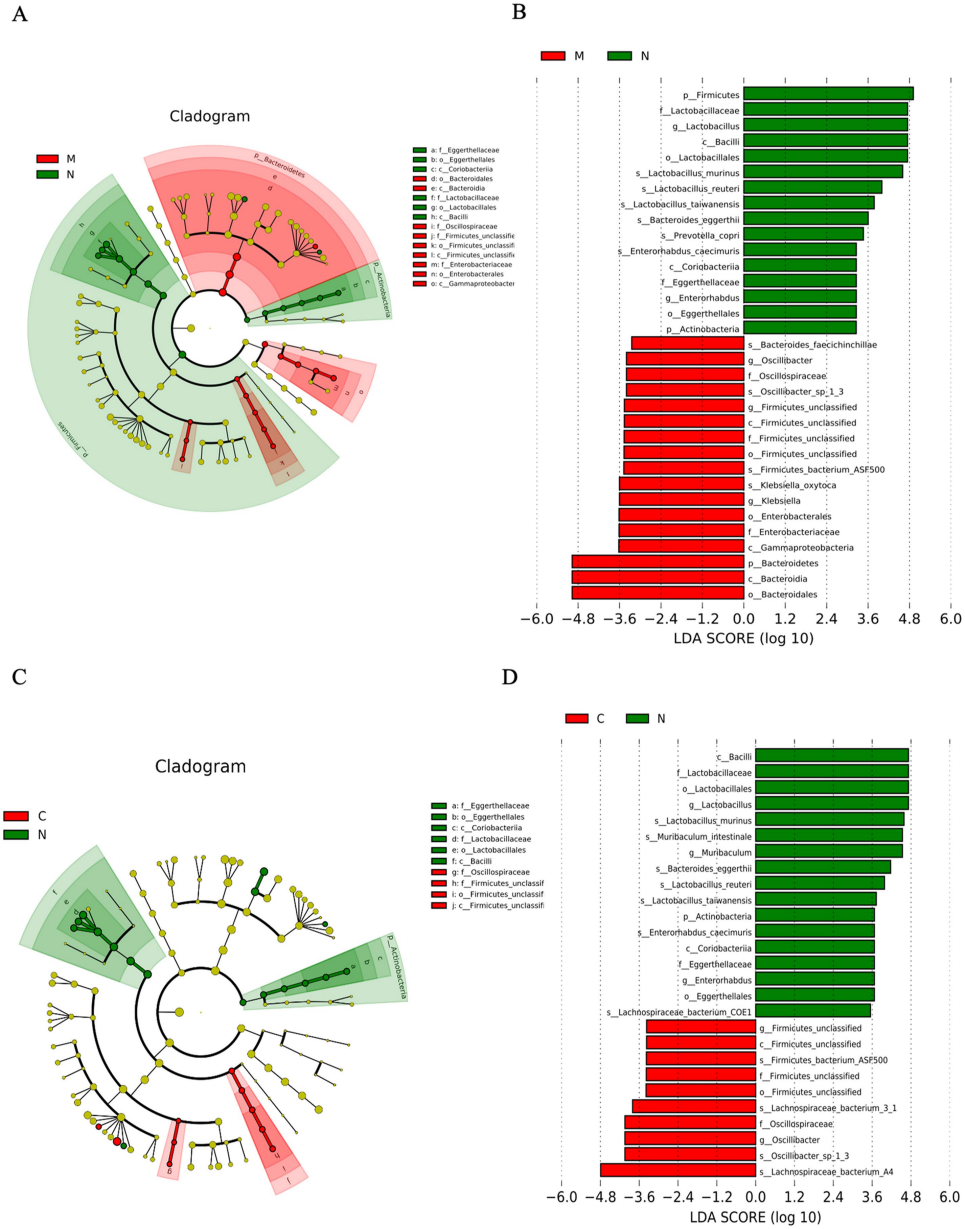
Differential analysis of gut microbiota composition between the relative groups. **(A)** Comparison of LEfSe cladograms between groups, illustrating taxonomic representation of differentially abundant bacteria between N and M groups. **(B)** Histogram of LDA scores from LEfSe analysis, showing the magnitude of effect size for differentially abundant taxa between N and M groups. **(C)** Comparison of LEfSe cladograms between groups, illustrating taxonomic representation of differentially abundant bacteria between N and C groups. **(D)** Histogram of LDA scores from LEfSe analysis, showing the magnitude of effect size for differentially abundant taxa between N and C groups.

**Figure 5:**
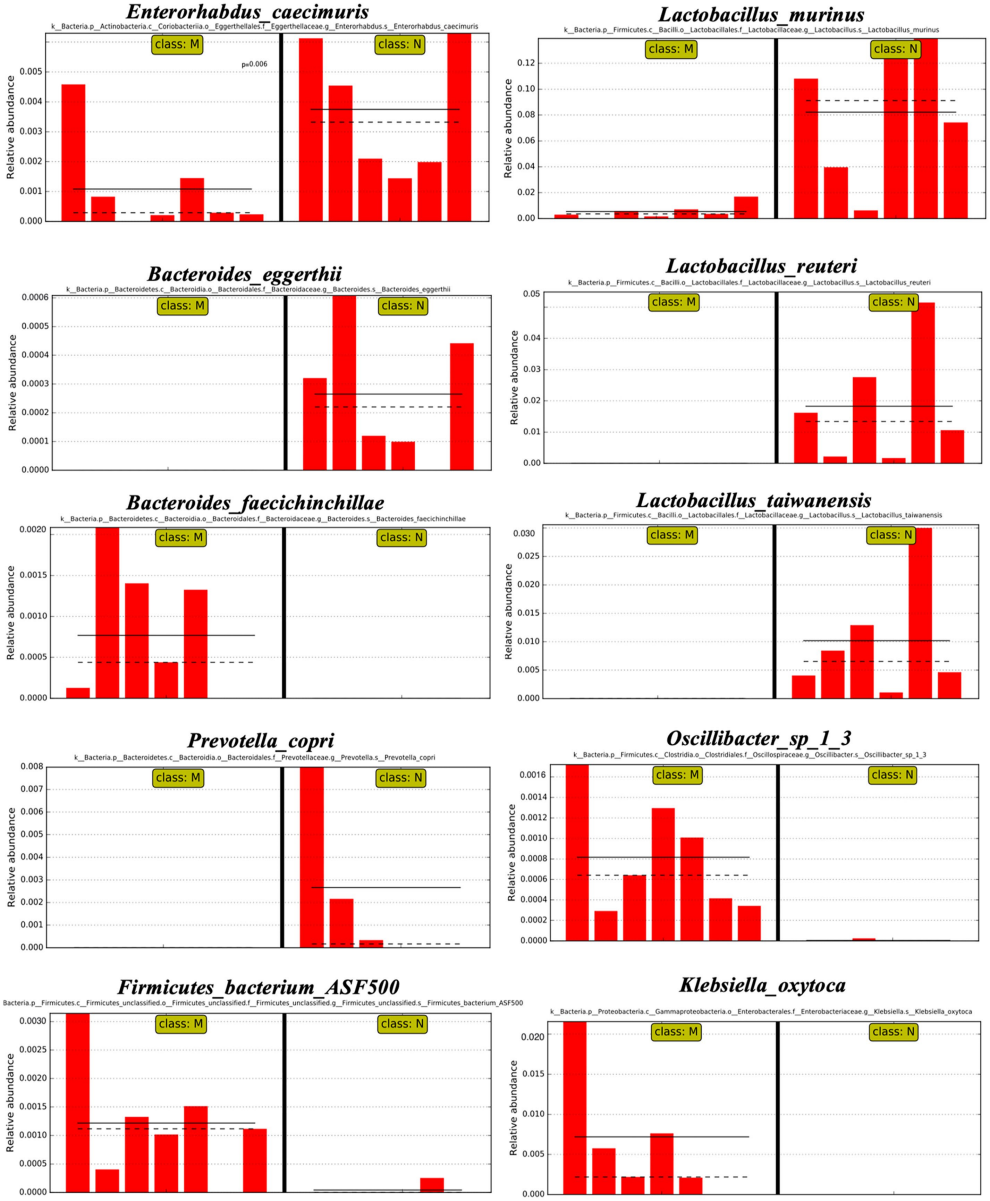
Differential analysis of gut microbiota composition between N and M groups. At the species level, there are significant strain differences between N and M groups. In the healthy group (N), the orders *Lactobacillales* and *Bacteroidales* predominated, whereas the M group exhibited altered abundance expression of various differential taxa. Specifically, the healthy group was characterized by *Lactobacillus murinus, Lactobacillus reuteri, Lactobacillus taiwanensis, an*d *Bacteroides eggerthii, wh*ile the *Parnassia* treatment group featured *Bacteroides faecichinchillae, Firmicutes bacterium* ASF*500*, *Oscillibacter* sp. *3_1*, *Klebsiella oxytoca, an*d *Enterorhabdus caecimuris*.

We further compared the gut microbiota between the M group and the healthy group. Differential analysis of gut microbiota composition between the N and M groups revealed significant strain differences at the species level (Figures 4A,B). In the healthy group (N), the orders Lactobacillales and Bacteroidales predominated, whereas the M group exhibited altered abundance of various differential taxa. Specifically, the healthy group was characterized by *Lactobacillus murinus*, *Lactobacillus reuteri*, *Lactobacillus taiwanensis*, and *Bacteroides eggerthii*, while the MHC treatment group featured *Bacteroides faecichinchillae*, Firmicutes bacterium ASF500, Oscillibacter sp. 3_1, *Klebsiella oxytoca*, and *Enterorhabdus caecimuris* (Figure 5). These findings suggest that orthotopic tumor implantation does indeed induce certain changes in the gut microbiota compared with healthy mice. Moreover, although oral administration of MHC extract inhibited tumor growth, some potentially pathogenic bacteria remained present.

### 3.4 Gut microbiota dysbiosis in an orthotopic brain tumor model

**Figure 6:**
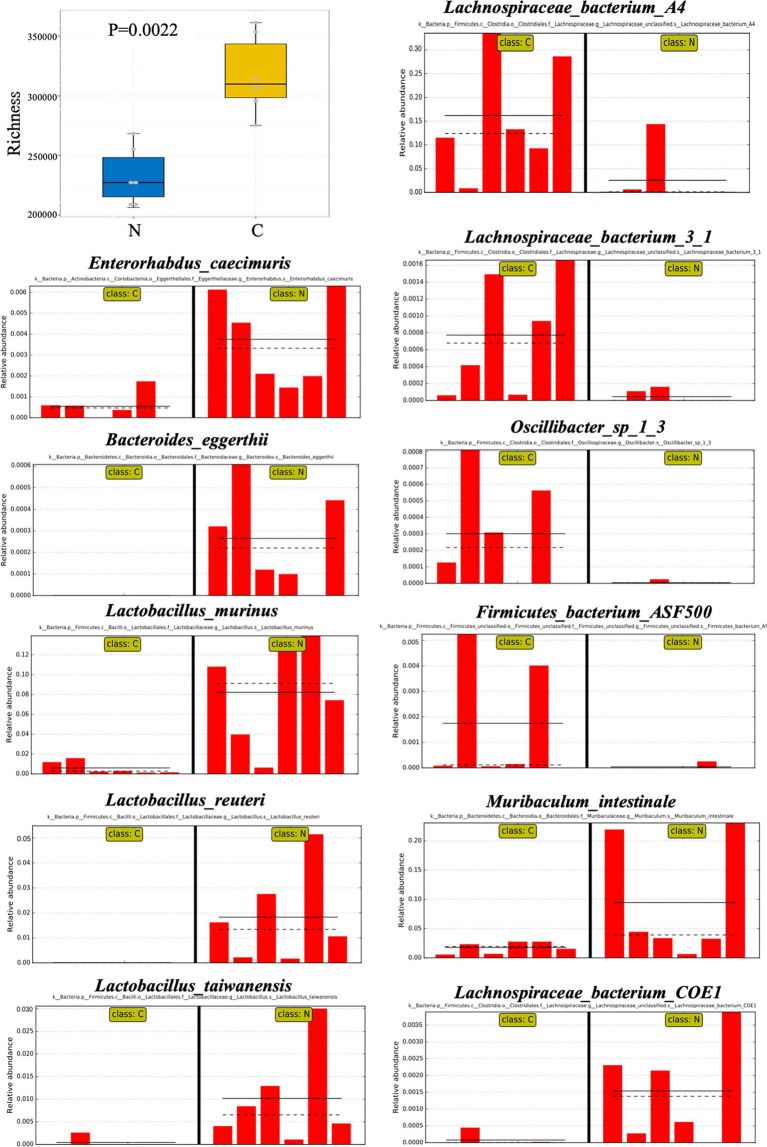
Differential analysis of gut microbiota composition and richness between N and C groups. At the species level, there were significant bacterial richness between N and C groups (*p* = 0.0022). In the healthy group (N), the orders *Lactobacillales* and *Bacteroidales* predominated, whereas the control group (C) was characterized by high abundance of the orders *Clostridiales* and *Lachnospirales*. Specifically, the healthy group was dominated by *Lactobacillus murinus, Lactobacillus reuteri, Lactobacillus taiwanensis,* La*chnospiraceae bacterium COE1*, *Muribaculum intestinale, an*d *Bacteroides eggerthii, wh*ile the model group (C) was dominated by *Firmicutes bacterium ASF500*, *Lachnospiraceae bacterium 3–1*, *Lachnospiraceae bacterium A4*, and *Oscillibacter* sp*. 1_3*.

To further evaluate whether orthotopic tumor-bearing mice exhibit alterations in gut microbiota, we conducted a comparison with healthy mice (Figures 4C,D). In the orthotopic tumor-bearing model, when evaluating the occurrence and progression of brain tumors, significant differences in the microbiota were observed compared to the healthy group. Among healthy mice (N), species such as *Lactobacillales*, *Muribaculum*, and *Enterorhabdu* were predominant. For instance, *Lactobacillus murinus*, *Lactobacillus taiwanensis*, *Lachnospiraceae bacterium COE1*, *Lactobacillus reuteri*, *Muribaculum intestinale*, *Bacteroides eggerthii*, and *Enterorhabdus caecimuris* showed significantly higher abundance in healthy mice compared to the model mice. In contrast, in the control model mice (C), *Firmicutes* and *Oscillibacter* were the most abundant genera, with *Firmicutes bacterium ASF500*, *Oscillibacter* sp. *1_3*, *Lachnospiraceae bacterium A4*, and *Lachnospiraceae bacterium 3_1* showing significantly higher abundance (Figure 6). These differential microbiota may be closely associated with the occurrence and progression of brain tumors and the metabolic regulation of the gut-brain axis.

## 4 Discussions

*P. palustris* Linn. (MHC) has a well-documented ethnopharmacological use against inflammatory diseases. Our study provides the first evidence that an MHC extract exerts anti-glioma effects *in vivo*, and that this activity is associated with significant modulation of the gut microbiota and the tumor immune microenvironment (TIME).

Gut microbiota shifts after MHC treatment. MHC treatment selectively enriched beneficial bacteria, notably *Muribaculum intestinale* and *Bacteroides faecichinchilla*, while reducing potentially harmful taxa such as Lachnospiraceae bacterium A4 and Helicobacter japonicus. The increase in *M. intestinale* is particularly relevant because this species produces butyrate and urocanic acid (UCA) – metabolites known to promote M1 macrophage polarization and to reduce MDSC recruitment via the CXCL1-CXCR2 axis, respectively (Zhang et al., 2025; Wang et al., 2026a; Bullock and Richmond, 2021; Marrocco et al., 2026). In line with the seminal findings of Bercik et al. (2011) demonstrating that gut microbiota independently influence central neurochemical and behavioral phenotypes, our microbial shifts provide a plausible link between MHC administration, enteric ecosystem remodeling, and CNS outcomes. Although we did not measure SCFAs or UCA directly, these microbial shifts are consistent with a gut-mediated anti-tumor mechanism.

Linking MHC compounds to microbial changes. Previous phytochemical studies have identified flavonoids (quercetin, kaempferol and their glycosides), phenolic acids (gallic acid), iridoids and saponins in MHC. Among these, quercetin and kaempferol glycosides are known prebiotics that enrich SCFA-producing bacteria including Bacteroides and Muribaculaceae (Li et al., 2024, Li X. et al., 2025; Shi et al., 2020; Majeed et al., 2025). Thus, the observed enrichment of *M. intestinale* and *B. faecichinchilla* is likely attributable to these flavonoid components. Concurrently, the reduction of Helicobacter and Lachnospiraceae A4 may result from direct antimicrobial effects of phenolic compounds or competitive exclusion.

Potential impact on the TIME and glioma progression. Butyrate from enriched *M. intestinale* can activate glycolysis in tumor-associated macrophages, shifting them toward an M1 anti-tumor phenotype (Yang et al., 2026; Wang et al., 2020). Additionally, Lactobacillus species (which were not significantly altered here, but they are known to produce indole-3-aldehyde) can drive CD8^+^ T cell differentiation via AhR activation (Montgomery et al., 2022; Bender et al., 2023). While we did not perform immune cell phenotyping in the TIME, the microbial changes we observed provide a plausible indirect mechanism for the reduced glioma growth. Direct anti-glioma effects of MHC compounds (e.g., quercetin-induced apoptosis via PI3K/Akt inhibition (Lou et al., 2016)) cannot be ruled out and may act synergistically with microbiota-derived signals. Our findings suggest that MHC extract suppresses glioma through a dual mechanism: direct cytotoxicity (likely mediated by flavonoids and phenolic acids) and indirect modulation of the gut microbiota and its metabolites, which in turn may reshape the TIME. Future work should include chemical fingerprinting of the extract, measurement of SCFAs/UCA, and immune profiling of the TIME to establish causality.

## 5 Limitations

The diverse array of compounds presents in *Parnassia palustris* (MHC)—including flavonoids (quercetin, kaempferol, and their glycosides), iridoid glycosides, phenolic acids (gallic acid), and saponins—collectively contribute to its anti-glioma effects through direct tumor inhibition, modulation of the gut microbiota, and regulation of innate immune components within the tumor microenvironment. The observed enrichment of beneficial taxa (*Muribaculum intestinale*, *Bacteroides faecichinchillae*) and suppression of potentially harmful bacteria (Lachnospiraceae bacterium A4, Helicobacter japonicus) following MHC treatment aligns with the known prebiotic and antimicrobial properties of these compounds. The convergence of butyrate production from Muribaculum and short-chain fatty acid (SCFA)-mediated M1 macrophage polarization provides a compelling mechanistic link between MHC-induced microbial shifts and glioma suppression in this model. However, no causal experiments (fecal microbiota transplantation, antibiotic-induced microbiota depletion, germ-free models, or metabolomic validation of microbial metabolites) were performed. Whether the observed microbiota changes directly mediate the anti-glioma effect—or are merely epiphenomena—remains unknown. Additionally, this study was conducted in Balb/c nude mice, which lack functional T cells. Therefore, our immune-related conclusions are restricted to innate immunity (macrophages, MDSCs, NK cells) and do not address T-cell-dependent anti-tumor responses. Future studies using immunocompetent syngeneic models (e.g., GL261 in C57BL/6 mice) are required to evaluate adaptive immune contributions to MHC’s anti-glioma effects. Another key design limitation of this study is the absence of a sham-operated group. The healthy control group (N) received no surgical manipulation, whereas both tumor-bearing groups (C and M) underwent craniotomy and intracranial tumor cell implantation. Consequently, we cannot definitively distinguish whether differences in gut microbiota between the healthy control and tumor-bearing groups arise from the tumor burden itself or from surgical stress (including craniotomy trauma, anesthesia, and potential intracranial pressure changes) (Albert et al., 2025; Danehower, 2021; Carabotti et al., 2015; Ma et al., 2019; Lin et al., 2025; Gu and Shu, 2024; Han et al., 2021). However, because both tumor-bearing groups (C and M) received identical surgical and anesthetic procedures, the observed microbiota differences between them (e.g., restoration effects following MHC treatment) are unlikely to be confounded by surgical factors. Importantly, our sampling time point (postoperative day 24) significantly exceeds the reported recovery window for surgery- or anesthesia-induced gut dysbiosis. Patrizz et al. (2020) found no significant *β*-diversity difference between sham-operated (craniotomy only) and healthy mice by day 15 post-surgery, and Han et al. (2021) showed that microbiota disturbances begin to recover by day 14. Thus, while the lack of a sham control limits direct comparison between healthy and tumor-bearing groups, the microbial changes observed at day 24 more likely reflect chronic tumor-induced pathophysiology than residual acute surgical effects. Future studies should include a sham-operated group and collect longitudinal samples to rigorously control for surgical confounding.

Moreover, we acknowledge that the present study, which relies on a crude extract of *Parnassia palustris* (MHC), has inherent limitations (Hu et al., 2013; Liu et al., 2023). The complexity of the extract makes it difficult to definitively pinpoint which specific compound(s) are responsible for the observed antitumor and microbiota-modulating effects. While the multi-component nature of the extract may contribute to its overall efficacy through synergistic interactions (Caesar and Cech, 2019), the lack of systematic fractionation and identification of the principal active constituents restricts our ability to establish a direct causal relationship between a single agent and the observed phenotype. Nevertheless, the chemical basis of MHC is not entirely undefined. Previous phytochemical investigations have shown that *P. palustris* (MHC) is rich in bioactive compounds, particularly flavonoids (such as quercetin, kaempferol, and their glycosides), phenolic acids (e.g., chlorogenic acid), and terpenoids (Xin et al., 2019; Hu et al., 2013; Liu et al., 2023). To further clarify the contribution of these components, we have already initiated systematic bioassay-guided fractionation (Aldrich et al., 2022). Our preliminary data indicate that flavonoid-enriched fractions exhibit potent anti-glioma activity *in vitro*, suggesting that these polyphenols may be key drivers of the extract’s efficacy (Wang R. et al., 2024; Seo and Yang, 2022). The comprehensive identification and validation of these active constituents will be the focus of our subsequent studies (Kong, 2025).

In the present work, we have chosen to focus on the holistic pharmacodynamic effects of the crude extract *in vivo* for two primary reasons. First, the study already encompasses a substantial body of work, including orthotopic tumor models, 16S rRNA sequencing, and metabolomics (Wang R. et al., 2024; Seo and Yang, 2022). Incorporating exhaustive phytochemical separation at this stage would unnecessarily diffuse the central research focus (Aldrich et al., 2022). Second, the crude extract is a more realistic representation of how the plant is used in traditional medicine (Liu et al., 2023), and its evaluation as a “prebiotic” aligns with the hypothesis that its beneficial effects on the gut microbiota are dependent on the synergistic action of multiple compounds (Caesar and Cech, 2019). As such, our findings provide a strong foundation for future studies aimed at deconvoluting the active principles of MHC and their mechanisms of action, which will be essential for translating these observations into a more defined therapeutic strategy (Kong, 2025; Aldrich et al., 2022).

## 6 Future directions

The present study establishes a significant association between MHC treatment, gut microbiota remodeling, and glioma suppression in an orthotopic mouse model. However, the causal relationship between these events remains to be definitively established (Kennedy et al., 2018). To rigorously test whether the antitumor effects of MHC are mediated by alterations in the gut microbiota, we plan to conduct antibiotic depletion experiments using a broad-spectrum antibiotic cocktail to deplete the endogenous microbiota (Reikvam et al., 2011; Panyod et al., 2026), followed by evaluation of whether MHC retains its antitumor efficacy. Concurrently, fecal microbiota transplantation (FMT) from MHC-treated donors into antibiotic-pretreated or germ-free tumor-bearing recipients will be performed to determine whether the microbiota alone is sufficient to transfer the antitumor phenotype (Bercik et al., 2011; Wouters et al., 2025). Furthermore, given that our study identified *Muribaculum intestinale* as a prominently enriched taxon following MHC treatment—and given that this species has been independently reported to negatively impact glioma growth in mice through TLR2-dependent immune activation, including enhanced pro-inflammatory profiles of tumor-associated microglial cells and increased CD8^+^ T cell frequencies (Marrocco et al., 2026)—we will prioritize single-strain colonization experiments with *M. intestinale* to verify whether this bacterium alone can recapitulate the tumor-suppressive effects of MHC. These causal validation experiments will constitute the core of our next-phase research.

Another critical direction for future investigation is to dissect the relative contributions of direct pharmacological actions versus microbiota-mediated indirect effects. While our *in vitro* data confirm that MHC extract and its flavonoid constituents (e.g., quercetin, kaempferol) exert direct cytotoxicity against glioma cells, and while chlorogenic acid and kaempferol have been shown to penetrate the blood–brain barrier, the extent to which these compounds reach the brain parenchyma *in vivo* after oral administration remains unknown. We therefore plan to perform systematic UPLC-Q-TOF-MS-based identification of MHC-derived compounds and their metabolites in plasma and brain tissues following oral gavage, which would provide direct evidence for brain penetration and central pharmacological activity (Wei et al., 2024; Wang Z. et al., 2024). Additionally, given the emerging role of short-chain fatty acids (SCFAs) such as butyrate in modulating the glioma immune microenvironment (Saadh et al., 2025; Chen M.L. et al., 2025), future metabolomic profiling of cecal contents and serum will be conducted to characterize microbiota-derived metabolites that may mediate the observed antitumor effects. The integration of causal microbiology experiments, pharmacokinetic analysis, and metabolomics will be essential to fully elucidate the dual direct and indirect mechanisms by which MHC exerts its anti-glioma activity.

## Data Availability

The data presented in the study are deposited in the NCBI Sequence Read Archive (SRA) repository, accession number PRJNA1503704.

## Glossary

BBB: Blood–brain barrier
CNS: Central nervous system
DAB: 3,3′-diaminobenzidine
DMEM: Dulbecco’s Modified Eagle Medium
FBS: Fetal bovine serum
FDA: U.S. Food and Drug Administration
FMT: Fecal microbiota transplantation
GBA: Gut-brain axis
GBM: Glioblastoma
H&E: Hematoxylin and eosin
HRP: Horseradish peroxidase
IHC: Immunohistochemistry
IOD: Integrated optical density
IVIS: *In vivo* fluorescence imaging system
LDA: Linear Discriminant Analysis
LEfSe: Linear Discriminant Analysis Effect Size
MAPK: Mitogen-activated protein kinase
MDSC: Myeloid-derived suppressor cells
MHC: *Parnassia palustris* (Meihuacao)
NK cells: Natural killer cells
NMDS: Non-Metric Multidimensional Scaling
NPs: Natural products
PBS: Phosphate-buffered saline
PCoA: Principal Coordinates Analysis
PI3K/Akt: Phosphatidylinositol 3-kinase / protein kinase B
RFP: Red fluorescent protein
SCFA: Short-chain fatty acid
TIME: Tumor immune microenvironment
TME: Tumor microenvironment
TMZ: Temozolomide
UCA: Urocanic acid
